# Walking on Visual Illusions

**DOI:** 10.1177/2041669520981101

**Published:** 2021-02-20

**Authors:** Greig Dickson, Daria Burtan, Shelley James, David Phillips, Jasmina Stevanov, Priscilla Heard, Ute Leonards

**Affiliations:** School of Psychological Science, University of Bristol, Bristol, United Kingdom; School of Fine Art, Royal College of Art, London, United Kingdom; Hemel Hempstead, United Kingdom; School of Psychological Science, University of Bristol, Bristol, United Kingdom; School of Psychology, University of the West of England, Bristol, United Kingdom; School of Psychological Science, University of Bristol, Bristol, United Kingdom

**Keywords:** architecture, bipedal gait, built environment, individual differences, perception/action, visual illusions

## Abstract

In nature, sensory and physical characteristics of the environment tend to match; for example, a surface that looks bumpy is bumpy. In human-built environments, they often don’t. Here, we report observations from people exploring if mismatch between visual and physical characteristics affected their perceived walking experience. Participants walked across four flat floors, each comprising of a visual illusion: two patterns perceived as alternating 3D “furrows and ridges,” the *Primrose Field* illusion, and a variant of the *Cafe Wall* illusion as a control pattern without perceived 3D effects. Participants found all patterns intriguing to look at; some describing them as “playful” or “gentle.” More than half found some of the patterns uncomfortable to walk on, aversive, affecting walking stability, and occasionally even evoking fear of falling. These experiences raise crucial research questions for the vision sciences into the impact of architectural design on well-being and walkability.

Human bipedal gait has evolved to allow us to travel long distances across the savannah and other landscapes, with our sensory systems predicting the physical characteristics of the environment through the sensory cues available (Gibson, 1979). In today’s built environments with new building materials and fashion trends toward the increased use of high-contrast repetitive patterns and striking perceptual effects, much of the sensory (in particular, visual) information picked up by our sensory systems can produce perceptions that diverge substantially from an accurate depiction of an environment’s physical characteristics. One of the most compelling examples of this is the glass skywalk of China’s Tianmen mountain park that stretches over 100 meters along the top of the Coiling Dragon Cliff. Whilst we might be rationally fully aware that the glass is physically stable and safe, the visual depth cues of the cliff drop below affect us more strongly than the visual cues of the glass surface, triggering in many people vertigo and automatic fear responses (see also the famous visual cliff experiments in babies by Gibson & Walk, 1960). Some less arresting whilst still eye-catching floor patterns (see Figure 1A to C for examples) in certain public squares and buildings contain illusory depth cues that might affect gait despite the floor being entirely flat (indeed, the corresponding author was alerted to such difficulties by comments from an older member of the public walking over the pattern shown in Figure 1B).

**Figure 1.**
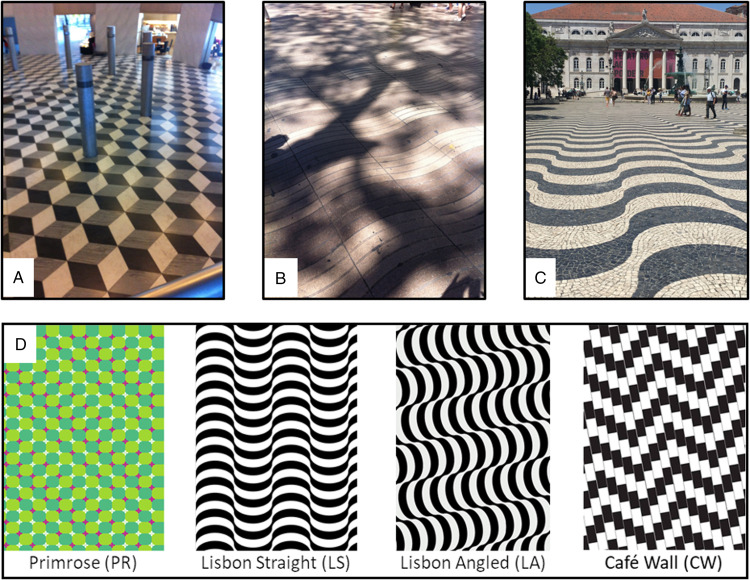
Real-world examples of floor patterns that induce 3D optical illusions: (A) Concert Hall, Kyoto, Japan; (B) La Ramblas, Barcelona, Spain; (C) Rossio Square, Lisbon, Portugal. (D) Schematic examples of the four floor patterns used in our study; PR optical illusion (Kitaoka, 2005, p. 22), LS and LA mimicking the pattern in Rossio Square on a smaller scale (i.e., higher spatial frequency); and the kindergarten pattern (Pierce, 1898) as a variant of the CW illusion (Gregory & Heard, 1979). Each floor consisted of the same vinyl material and was 6 m long and 1.50 m wide. Floors were fixed to the ground with black 10-cm wide duct tape along all sides. PR = Primrose; LS = Lisbon Straight; LA = Lisbon Angled; CW = Café Wall.

There is no doubt that visual illusions intrigue young and old alike (Shapiro & Todorovic, 2017), and the fashion industry draws from this (Elshafei, 2015). Moreover, the study of visual illusions has a long tradition in the visual sciences as it provides a powerful tool to gain insights into the mechanisms underlying visual perception (for reviews, see Carbon, 2014; Shapiro & Todorovic, 2017). Yet, little is known about how the mismatch between visual and proprioceptive characteristics of floor patterns on a larger scale impacts perceived walking experience and actual gait kinematics. For an ageing population, such understanding is particularly important to ensure the accessibility and inclusivity of our environments.

Before investing into a complex, fully controlled research study, we designed a Public Engagement activity to capture people’s experiences when walking on perfectly flat vinyl floors containing visual illusion patterns (see Figure 1D). This activity was run in the context of Public Engagement events within a Bristol (UK) community (*n* = 49) and alongside two scientific conferences (6th Visual Science of Arts Conference, and 41st European Conference on Visual Perception) in 2018 (*n* = 97) at events open to the general public. Illusions included two black and white patterns perceived as “wavy,” with alternating 3D “furrows and ridges” (named *Lisbon Straight* and *Lisbon Angled* after the tiling patterns in Rossio Square in Lisbon, Portugal; Figure 1C); the coloured *Primrose Field* illusion (Kitaoka, 2005, p. 22); and a *kindergarten pattern* first described by (Pierce, 1898) as a variant of the *Cafe Wall* illusion (Gregory & Heard, 1979). Note that this latter illusion was intended to serve as a kind of high-luminance contrast, high spatial frequency control pattern without 3D effect. Indeed, many participants unfamiliar with the Café Wall illusion did not realise that this pattern was an illusion in its own rights. Participants walked across each floor in the order they preferred and then compared the perception of illusion strength for the four floors with each other and rated each floor separately for its walking comfort. In addition, free text comments were collected to evaluate the quality and breadth of experiences of walking over each pattern (Supplemental Material).

Figure 2A and B shows group averages for (a) perceived relative illusion strength when comparing the four floors with each other (if participants saw no illusion on a given floor, they rated this pattern as 0) and (b) walking discomfort ratings for each floor, respectively. The Lisbon Straight pattern evoked the strongest illusion, followed by the Lisbon Angled pattern, the Primrose pattern, and then the Café Wall pattern. The latter did not evoke any illusion in about 15% of participants (ranking of 0). Differences in perceived relative illusion strength were significant as confirmed by a one-way repeated measures analysis of variance, *F* (3, 536) = 75.44, *p* < .001, partial $\eta^{2}$ = .360; all post hoc Tukey comparisons were significant at the *p* < .01 level or more.

**Figure 2.**
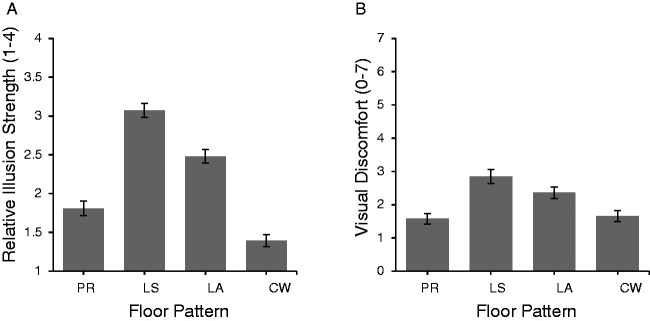
(A) Group average rankings for relative perceived illusion strength (rankings between 1 and 4; note that for CW, 15% of answers were 0 as participants did not perceive these patterns as illusions) and (B) group average ratings for perceived walking discomfort (ratings from 0 to 7) for each of the four floor patterns. Error bars represent ± 1 SEM. PR = Primrose; LS = Lisbon Straight; LA = Lisbon Angled; CW = Café Wall.

Similarly, the highest walking discomfort was reported for the Lisbon Straight pattern, followed by the Lisbon Angled pattern, whilst both the Primrose and the Café Wall patterns were perceived as similarly (un)comfortable to walk on. A second one-way repeated measures analysis of variance confirmed that perceived walking discomfort differed significantly between all pattern types, *F* (3, 552) = 21.89, *p* < .001, partial $\eta^{2}$ = .137. Post hoc Tukey tests revealed that apart from the comparison between the Café Wall and the Primrose pattern and between the two Lisbon patterns, respectively, all other comparisons were significant (*p* < .05).

Walking discomfort thus seems to at least partially mirror perceived (ranked) illusion strength results. Moreover, relative illusion strength and perceived walking discomfort correlated for each of the four floor patterns—Primrose *r* (138) = .42, *p* < .001; Lisbon Straight *r* (138) = .26, *p* < .005; Lisbon Angled *r* (138) = .23, *p* < .01; Café Wall *r* (133) = .31, *p* < .001. Note, however, that our questionnaire design prevents us from excluding the possibility that some of the participants realised that we were expecting a relationship between illusion strength and walking discomfort.

A closer look at the distribution of walking discomfort ratings, however, revealed substantial individual differences (see Figure 3): A large proportion of participants reported no discomfort at all when walking over the floors (almost half of all participants for the Primrose pattern, about 40% for the Café Wall, and almost 30% for the two Lisbon patterns). For the remaining participants, walking discomfort ratings varied widely across patterns, from slight discomfort to strong aversive reactions as confirmed by qualitative comments.

**Figure 3.**
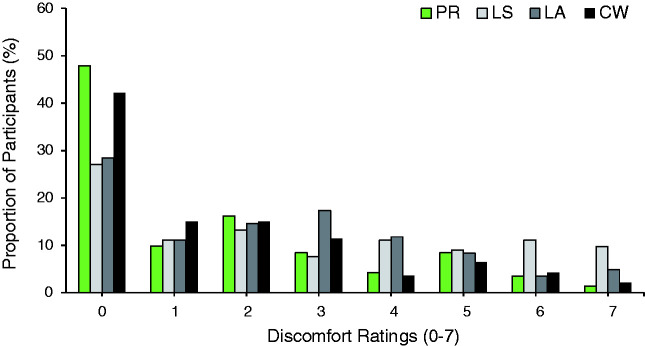
Individual differences for perceived walking discomfort. The figure shows the proportion of participants (in %) per discomfort point for each of the four floors. Green: PR; light grey: LS; dark grey: LA; and black: CW. PR = Primrose; LS = Lisbon Straight; LA = Lisbon Angled; CW = Café Wall.

Qualitative comments fell into three subthemes: perception, walking experience, and emotional response (Figure 4).

**Figure 4.**
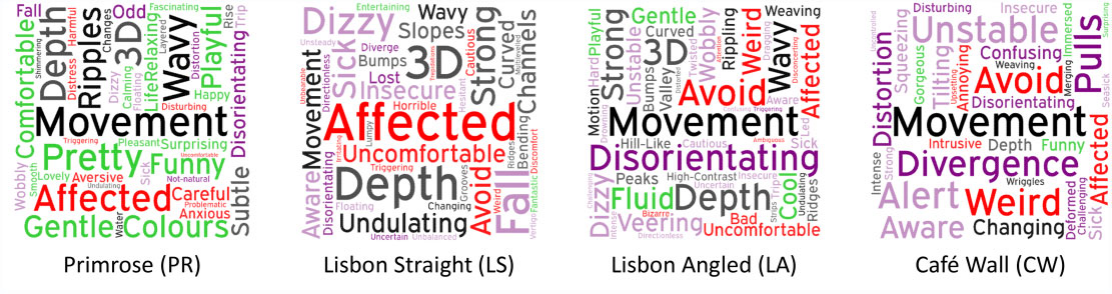
Most commonly occurring words in participants’ statements referring to their experience of walking on each of the patterns. Word size is determined by the amount of times it occurred. Word colour is determined by the subthemes. Perceptual illusions: perception of depth (dark grey), perception of movement (black). Walking experience: disorientation (dark purple), perceived instability (light purple). Emotional response: negative feelings (red), positive feelings (green). PR = Primrose; LS = Lisbon Straight; LA = Lisbon Angled; CW = Café Wall.

## Perception

Participants reported to have perceived depth not only whilst looking at but also whilst walking over the Primrose, Lisbon Straight, and Lisbon Angled patterned floors. For both Lisbon patterns, floors were perceived as three-dimensional, rising up and down in “ridges” or “bumps” as one would expect for geometrical illusions. The Primrose pattern was described as appearing to “ripple” and “shimmer,” in line with earlier descriptions of combining geometrical and motion illusion effects. Walking over the floors evoked additional sensations of *movement*; the Primrose pattern was referred to as dynamically “wavy,” providing a gentle sense of *movement* like walking over a meadow or on water. The “ridges” and “furrows” of the Lisbon patterns were described as dynamically moving up and down in the direction of their respective orientations, sometimes seemingly reversing height for the Lisbon Angled pattern during the walk. Even the Café Wall pattern was perceived as “weaving” with its parallel lines dynamically converging and diverging as participants walked.

## Walking Experience

Lisbon Angled, Café Wall, and Primrose patterns further affected participants’ perceived ability to walk in a straight line, evoking a sense of being “pulled”/veering to the left in the main direction of the patterns. This perceived veering mirrors the objectively measured veering when people walk over a floor with oblique lines (Leonards et al., 2015). Perceived veer is thus most probably related to the high-contrast oblique patterns rather than the presence of illusions. More interesting was the description of the two Lisbon patterns as “disorientating”: particularly older participants—irrespective of whether participating at the community event or at activities in the context of the Science Conferences—said they felt uncertain of the height of the floor surface and where to place their feet in relation to the patterns. For the Lisbon Straight pattern, a quarter of participants felt uncomfortable and uncertain whether to place their feet “within a furrow” or “on top of a ridge,” with 16 participants stating that they intentionally walked on the “ridges” of the two Lisbon patterns to account for the ambiguity of the perceived surface level.

In addition to walking discomfort, participants reported feelings of increased instability, expressed in words such as “unsteady,” “unstable,” “uncertainty,” “need to walk more slowly,” “walk more carefully,” “walking instability,” and “feeling dizzy”.

## Emotional Response

Most participants described negative feelings when walking over the high-contrast Lisbon and Café Wall patterns, even though participants generally agreed that the patterns per se were intriguing. Some participants even commented for the Lisbon patterns that, in the real world, they “would avoid looking at”/”walking on such patterns” or that they found the walking experience “horrible” or “uncomfortable”. Participants were far more likely to describe walking on the Primrose pattern as a walking experience they enjoyed, with statements such as “pleasant movement, relaxing and comfortable”, “like walking on water”, “like gliding over the floor”, or wanting “to dance and play on it”. Several participants even wondered whether the Primrose patterns were printed on a softer, more padded material than the other floors.

Overall, this exploration and the differences in experiences it provoked for different patterns suggest that walking over floors containing high-contrast patterns such as the visual illusions used here might affect people’s walking experience -- often, but by no means always, in a negative way. The lack of adequate control floors without illusions does not allow us to disentangle how much of these effects described here were due to the presence of illusions per se, the specific type of illusion or how much was simply the effect of high-contrast patterns. Nor can we draw conclusions about how reported effects were impacted by the exact environment, the speed with which people walked, where participants looked relative to the patterns, how quickly they adapted to the floors, or whether they would have felt similar effects without a perceptual scientist asking questions.

Despite the study’s obvious limitations, we feel encouraged to call for a new line of research into the parameters that underpin the link between floor pattern characteristics and human gait, and perception of instability of the walking surface. In particular, the impact of the degree of perception of depth and movement of patterns in designed walkways should be investigated further, in relation to feelings of disorientation and instability, positive experiences, and how such experiences are related to quantifiable adaptations of gait kinematics themselves. Recent evidence supports the notion of a direct impact of floor pattern on gait kinematics: Certain aspects of floor patterns (such as the orientation of tiling or the spatial frequency of stripes) have been shown to influence locomotion characteristics, such as lateral veer (Leonards et al., 2015) and walking speed (Ludwig et al., 2018). In addition, perceived scene motion has been shown to modulate horizontal trunk displacement (Logan et al., 2010), suggesting a decline in stability during bipedal locomotion. This might affect the responses in leg kinematics, which, in turn, would disrupt the gait cycle and lead to walking instability (Frost et al., 2015). Other research might want to take an approach less common to the perception sciences by collecting data of people walking over existing patterned floors in the real world—both with and without visual illusions—using CCTV footage to measure changes in gait. Moreover, one could investigate whether problems have been reported to authorities about particularly striking floor patterns in public spaces.

Why should vision scientists care? As a result of the evolution of architectural design and increases in modular design, high-contrast and repetitive patterns are much more pervasive in urban environments (Wilkins et al., 2018), where increasingly more of the global population live (Park & Burgess, 1925). Architectural design choices, as well as solutions to practical problems, (such as, e.g., for barrier matting; Harle et al., 2006), have also increased the amount of visual illusions that are present in urban environments (Penacchio & Wilkins, 2015). To date, vision research has paid little attention to how such patterns impact the way we move and, consequently, feel in everyday life contexts. Given the increasingly aging population which is much more reliant on visual information for postural control (Li et al., 2018), it seems crucial to understand how visual aspects of the built environment impact on our walking behaviour.

Last, but not least, do we think that the original *Lisbon* pattern in Lisbon’s Rossio Square (see Figure 1C) negatively affects people’s gait? Most likely not: Lisbon’s Rossio Square pattern consists of far lower spatial frequencies and luminance contrasts than the ones used in our design. Yet, this remains to be tested.
